# Oral Manifestations of Varicella and Their Contribution to Clinical Assessment in Hospitalized and Outpatient Patients

**DOI:** 10.3390/life16040673

**Published:** 2026-04-15

**Authors:** Velina Stoeva, Veselina Kondeva, Rumyana Stoyanova

**Affiliations:** 1 Department of Epidemiology and Disaster Medicine, Faculty of Public Health, Medical University of Plovdiv, 4002 Plovdiv, Bulgaria; velina.stoeva@mu-plovdiv.bg; 2 Department of Pediatric Dentistry, Faculty of Dental Medicine, Medical University of Plovdiv, 4002 Plovdiv, Bulgaria; veselina.kondeva@mu-plovdiv.bg; 3 Department of Health Management and Health Economics, Faculty of Public Health, Medical University of Plovdiv, 4002 Plovdiv, Bulgaria

**Keywords:** chickenpox, oral cavity changes, enanthema

## Abstract

Background: Oral lesions, particularly enanthema, may accompany chickenpox and represent an important but often underrecognized component of the clinical presentation. Their timely identification is especially relevant in dental practice, as oral manifestations may be more frequent in patients with a more severe clinical course. This study aimed to describe characteristic oral cavity changes in hospitalized and outpatient patients with chickenpox, to identify patterns in the occurrence of oral findings in relation to disease severity, and to support clinical assessment in practice. Methods: A retrospective review of medical records was conducted for patients diagnosed with chickenpox in Bulgaria between December 2023 and May 2025. Data from hospitalized patients and outpatient cases were analyzed and compared to evaluate the distribution of oral manifestations and their association with clinical severity. Results: A total of 144 patients were included, of whom 32.6% required hospitalization. Oral enanthema was more frequently observed among hospitalized patients (48.8%). In univariate analyses, oral enanthema and tongue changes were associated with hospitalization. Multivariable logistic regression identified age and body temperature as independent factors associated with hospitalization, while oral manifestations did not retain independent predictive significance. Conclusion: Oral enanthema was more frequently observed among hospitalized patients and was associated with a more severe clinical presentation in univariate analyses. Although oral findings should not be interpreted as independent predictors of disease severity, their recognition may support clinical assessment, dental treatment planning, and appropriate infection control measures.

## 1. Introduction

Varicella is characterized by a pseudopomphoid rash of macules, papules, and vesicles, with lesions at different stages appearing simultaneously. Some patients also develop lesions on the oral mucosa, known as enanthema, which often precedes or occurs in parallel with the classic exanthema. Oral manifestations include vesicles, ulcers, and lesions on the palate, buccal mucosa, gums, and, in some cases, the tongue. These oral symptoms have important diagnostic significance and should be recognized by dentists, as they may be among the first healthcare professionals to identify the infection, which is important for early recognition, timely treatment of lesions, prevention of complications, and a multidisciplinary approach to patient care. The severity and number of oral lesions correlate with the severity of the clinical course: in severe cases, lesions are usually present and more numerous, while in milder cases, oral manifestations are reported significantly less frequently [1,2]. Knowledge of the oral manifestations of varicella is crucial for timely diagnosis and is part of the dentists’ responsibilities in their collaboration with pediatricians and infectious disease specialists, to improve the quality of interdisciplinary care and emphasize the importance of an integrated approach to patient management [1,2,3].

Varicella is transmitted by inhalation of contaminated aerosol droplets from infected individuals, making it highly contagious in dental offices. Thus, the entire dental clinic can be contaminated, not just the area around the dental office [1,2]. Dentists must be able to recognize the infection and implement strict infection control measures to limit transmission to other patients and staff.

In children, the first symptom is often a rash in the mouth. The most common visible manifestations appear on the gums, buccal mucosa, and tonsils, sometimes involving the pharyngeal mucosa, in the form of small ulcers that may be painful or pruritic. These symptoms appear 11 to 21 days (mean 14 days) after infection [4]. The number of oral lesions can vary considerably, from 1–2 to more than a dozen. The dynamics of the oral rash (macules, vesicles, shallow ulcers) is similar to that of the skin, with lesions appearing in waves rather than simultaneously [5,6,7]. Oral findings may be associated with varicella and may aid in the clinical recognition and assessment of the disease but should not be interpreted as independent predictors of disease severity.

Despite the clinical importance of oral manifestations in varicella, data on the incidence and clinical significance of oral manifestations in hospitalized versus outpatient cases remain limited, particularly from a dental perspective.

This retrospective epidemiological study aimed to describe characteristic oral cavity changes in patients with chickenpox, including those hospitalized in the Infectious Diseases Clinic and registered outpatient cases, to identify patterns in the manifestation of oral findings in relation to the clinical course of chickenpox, and to support clinical assessment and management.

## 2. Materials and Methods

### 2.1. Procedure

The medical records of patients hospitalized in an Infectious Diseases Clinic in a large regional city in Bulgaria were reviewed. The study covered a period of approximately one and a half years, from December 2023 to May 2025, chosen due to the pronounced winter–spring seasonality of varicella. Data were collected retrospectively from patients diagnosed with chickenpox using a checklist developed specifically for this study (Supplementary File S1). The checklist included demographic variables (age, sex), clinical variables (maximum recorded body temperature, categorized temperature severity, type of skin rash, oral cavity findings, tongue status), and outcomes (hospitalization status).

Medical records from prehospital care in pediatric outpatient practices covering the same period were also analyzed, including only patients with a confirmed diagnosis of varicella. To ensure an adequate number of outpatient cases for comparison with hospitalized patients, two pediatric outpatient practices were randomly selected from the registry of the Regional Health Insurance Fund (RHIF)–Plovdiv using simple random sampling (random number generator). Both practices served the same geographic area and study period as the hospitalized cases. Permission to participate in the study was obtained from the practice managers, and the medical records of patients who had recovered from chickenpox were made available for analysis.

All retrospectively reviewed patients included in this study had a confirmed diagnosis of varicella (chickenpox). Inclusion criteria were: (1) patients hospitalized with a diagnosis of varicella in an Infectious Diseases Clinic, and (2) patients diagnosed with varicella in prehospital outpatient pediatric care during the same study period. Exclusion criteria were individuals without a confirmed diagnosis of varicella. The analyzed dataset included all registered patients with varicella from the participating healthcare institutions during the study period.

Comorbidities were recorded during data extraction; however, due to their low frequency among hospitalized patients and inconsistent documentation, they were not included in the statistical analysis. The reported comorbid conditions in individual hospitalized patients included chronic obstructive pulmonary disease, arterial hypertension, thalassemia minor (after total hysterectomy), congenital heart defects (interventricular septal defects, coarctation of the aorta, mitral stenosis), toxic drug effects, and acute laryngitis. These conditions were not associated with subsequent complications related to varicella in the respective patients. No comorbidities were reported among outpatient cases.

Complications were observed in a limited number of patients and included pneumonia, skin abscesses, carbuncles, furuncles, exacerbation of chronic obstructive pulmonary disease, skin infection, and one case of pneumonia in a pregnant woman in the tenth gestational week.

According to the available medical records, no concomitant oral infections were reported in either patient group that could have influenced the extent or severity of oral manifestations. The oral enanthema described in this study represents a typical manifestation of varicella caused by varicella-zoster virus infection and is not characteristic of odontogenic or tooth-related infections.

Treatment of varicella was primarily symptomatic, with antiviral therapy (acyclovir) administered in severe cases according to standard clinical practice and is not expected to influence the presence or characteristics of oral manifestations.

The study complied with established standards and adhered to the requirements of the Declaration of Helsinki on ethical principles for medical research and the principles of Good Clinical Practice. It was approved by the Research Ethics Committee at the Medical University of Plovdiv (Opinion P-KHE-27/6 March 2026).

Data were obtained through a review of medical records of hospitalized and outpatient patients diagnosed with chickenpox during the study period. For data protection purposes, all patients were assigned numeric codes when completing the registration forms for oral and general clinical manifestations, and the extracted information could not be directly linked to individual patients.

### 2.2. Data Analysis

The data were processed and analyzed using IBM SPSS Statistics, version 23.0. Descriptive statistics were performed to characterize the sample; mean values (Mean) and standard deviations (SD) were calculated for quantitative variables, and frequencies and percentages for categorical variables.

To assess statistical associations between categorical variables, the chi-square (χ^2^) test and Fisher’s Exact Test were used. To compare mean values between two independent groups, Welch’s *t*-test for independent samples was applied. This test was chosen due to unequal variances and its robustness to moderate deviations from normality.

To identify factors associated with the probability of hospitalization, binary logistic regression analysis was performed. Variables showing statistical significance in univariate analyses were included in the model. The results are presented as regression coefficients (B), standard errors (S.E.), Wald statistics, degrees of freedom (df), *p*-values, and odds ratios (Exp(B)) with 95% confidence intervals. The level of statistical significance was set at *p* < 0.05.

## 3. Results

The total number of patients included in this retrospective study was 144, distributed as follows: 32.6% (*n* = 47) were hospitalized, and 67.4% (*n* = 97) received pre-hospital care. The mean age of the overall sample was 9.19 ± 11.66 years, ranging from 12 days to 66 years. This wide variability reflects a skewed age distribution, with a higher proportion of adult patients in the hospitalized group (mean age 16.45 ± 17.94 years) compared to the outpatient group (mean age 5.67 ± 3.15 years). The proportion of men was 56.3% (*n* = 81), and that of women was 43.8% (*n* = 63). Table 1 presents the distribution of clinical manifestations in the total sample.

As shown in Table 1, a significant proportion of patients were subfebrile (41.0%, *n* = 59), with high fever observed in 4.9% (*n* = 7), and hyperpyrexia recorded in only one patient (0.7%). The rash in chickenpox was maculo-papulo-vesicular, sometimes described as a “starry sky,” and was observed in most patients (78.5%, *n* = 113). The presence of enanthema in the oral cavity is among the characteristic oral manifestations of chickenpox and has important diagnostic significance, occurring in 28.5% of patients (*n* = 41). A hyperemic throat was also frequently observed, reported in 88.9% (*n* = 128) of all patients.

The tongue was normal and not coated in most patients (67.4%, *n* = 97). These findings are consistent with literature reports: in chickenpox, changes in the oral mucosa, including the tongue, can occur, but they are not the most common site, with lesions more frequently observed on the lips, palate, and buccal mucosa [8].

Table 2 presents the demographic and clinical characteristics of the two patient groups and their relationships with the type of treatment.

As shown in Table 2, the non-parametric analysis (Fisher’s Exact Test, *p* = 0.282) did not reveal a statistically significant difference in the proportion of men and women treated in outpatient or inpatient settings.

The association between higher recorded temperature values and the need for hospitalization, reflecting more severe clinical conditions, was expected (*p* < 0.001) and is clearly observed in Table 2.

The association between characteristic changes in the oral cavity-enanthema-and the need for hospitalization (*p* = 0.010) is notable. Oral enanthema was significantly associated with hospitalization in univariate analyses, indicating that oral manifestations occurred more frequently in patients with a more severe clinical course. Among hospitalized patients without enanthema, 25.5% (*n* = 26) required hospitalization, whereas this proportion increased to 48.8% (*n* = 20) among patients with enanthema. The only patient with aphthae in the oral cavity also required hospital treatment.

Findings regarding the tongue were similarly informative. Among patients with no tongue changes, 26.8% (*n* = 26) were hospitalized, whereas hospitalization occurred in 34.6% (*n* = 9) of those with a dry, coated tongue, and 57.1% (*n* = 12) of patients with a moist, coated tongue. Thus, a moist, coated tongue was more frequently observed among hospitalized patients and was associated with a more severe clinical course in univariate analysis (*p* = 0.026).

Average age also influenced the probability of hospitalization, with higher age associated with increased likelihood of inpatient care (*p* < 0.001).

To assess the combined influence of factors associated with treatment type (outpatient versus inpatient), binary logistic regression was performed. Variables that were statistically significant in univariate analyses (oral enanthema and tongue findings) were initially entered into a multivariable logistic regression model together with age and body temperature. However, these oral variables did not retain statistical significance in the multivariable context and were associated with sparse data in some categories, resulting in unstable estimates. Therefore, a simplified and more robust final model including only age and body temperature was retained and presented. The final model was statistically significant (Omnibus Tests χ^2^ = 48.352, df = 2, *p* < 0.001) and explained 39.8% of the variance in hospitalization status.

As shown in Table 3, both age and body temperature were independently associated with hospitalization. Each additional year of age was associated with a 12.3% higher probability of hospitalization compared to outpatient management (Exp(B) = 1.123; *p* < 0.001). For patients of the same age, an increase of one level in body temperature category (e.g., from afebrile to subfebrile or from febrile to high fever) was associated with an approximately 2.8-fold higher likelihood of hospitalization (Exp(B) = 2.845; *p* < 0.001).

## 4. Discussion

Oral symptoms of varicella are important for the timely recognition of the infection, as they may sometimes appear before the exanthema and typically present as enanthema, often described as small blister-like formations covering different areas of the oral mucosa and clinically resembling the vesicles of primary herpes simplex virus. These intraoral vesicles are observed on the tongue, oral mucosa, gums, palate, and oropharynx and are generally not very painful [9].

In our study, the absence of oral enanthema was observed in 25.5% (*n* = 26) of hospitalized patients and in 74.5% (*n* = 76) of outpatients. Overall, oral enanthema was identified in 48.8% (*n* = 20) of hospitalized patients, while aphthous lesions were recorded in 2.1% (*n* = 1) of cases. Despite the relatively small sample size and the limitations inherent to the retrospective design, such as reliance on previously recorded data, potential underreporting of oral findings in outpatient settings, and lack of dynamic follow-up, our results demonstrate an association between the presence of oral enanthema and disease severity in univariate analyses. Oral manifestations were observed more frequently in patients with a more severe clinical presentation; however, they were not independent predictors of hospitalization. Nevertheless, their recognition remains clinically relevant, particularly for raising awareness of potentially more severe cases, informing dental treatment planning, supporting appropriate infection control measures in dental practices, and facilitating timely referral to medical specialists.

It is important, in diagnostic terms and for correct differential diagnosis, to be familiar with the oral manifestations of herpes zoster. It occurs more often in older or immunosuppressed patients, but not exclusively, and it is important to distinguish it from primary varicella infection. In it, a prodromal stage of pain precedes visible lesions by several days. Oral manifestations of the acute phase begin as vesicles over erythematous macules that ulcerate and crust over approximately 10 days, affecting both keratinized and nonkeratinized mucosa. Postherpetic neuralgia may follow, resulting in severe pain that can last a year or longer [10,11,12,13].

Oral enanthema precedes the exanthema on the body and is described as prominent vesicle-like lesions that persist for about a day before progressing to shallow, yellow or gray ulcers, which are not crusted [4]. Similar changes were observed in our patients, with vesicular lesions appearing in parallel with or preceding each skin rash and persisting for 1–2 days.

The number and distribution of oral lesions have been shown to correlate with disease severity. Studies report that in mild clinical forms, oral lesions are observed in approximately one third of cases, usually as one or two lesions resolving within 1–3 days, while in severe cases, oral lesions can persist for 5–10 days [14]. Our data are consistent, as we found a statistically significant association between the presence of oral enanthema and the need for hospitalization (*p* = 0.010).

Another study also supports this correlation: the severity of general clinical status correlates with the number of oral lesions. Over 3 years, 62 children and one 4-year-old with herpes zoster of the third branch of the trigeminal nerve were examined. In group B (17 severe cases), oral manifestations were always present, with 5–30 lesions on the oral cavity, lips, palate, cheek, buccal mucosa, and tongue. In group B (26 moderately severe cases), oral lesions were observed in 23 cases, with 2–10 lesions located on the lips, palate, cheek, gums, and tongue. In group A (19 mild cases), oral lesions were present in only 6 cases, with 1–2 lesions localized on the lips, hard and soft palate, and gingiva [8].

Another study of characteristic oral presentations of various infections, including varicella, emphasized the need for pediatric dentists to diagnose and manage common oral manifestations and to refer children to pediatricians when systemic involvement is suspected [15]. Several publications suggest that the frequency and severity of oral lesions may correlate with the overall severity of varicella infection. In more severe forms, more extensive, painful ulcerations can interfere with feeding and hydration, increase the risk of secondary bacterial infection, and prolong hospital stay. These observations support the value of interdisciplinary involvement of dentists and pediatricians in patient care [16].

For correct differential diagnosis, it is crucial to distinguish oral manifestations of VZV infection from other viral infections with similar oral findings, such as herpes simplex virus (HSV) and human papillomavirus (HPV) [17]. According to another study, herpesvirus-related infections affect 60–95% of adults and include gingivostomatitis, orofacial and genital herpes, as well as primary varicella and herpes zoster. Symptoms, treatment, and potential complications vary depending on primary or recurrent infection and the patient’s immune status [18].

Reported complications after varicella include encephalitis, pneumonia, Reye’s syndrome, and Guillain–Barré syndrome [19]. The most common complication of VZV reactivation is postherpetic neuralgia, although a range of neurological syndromes can occur, including vasculitis, encephalitis, segmental motor weakness, myelopathy, cranial neuropathies, and Guillain–Barré syndrome [20]. In our study, complications were observed in a limited number of patients, including pneumonia, skin abscesses, carbuncles, furuncles, exacerbation of COPD, skin infection, and one case of pneumonia in a pregnant woman in the tenth week of gestation.

Although oral manifestations were associated with hospitalization in univariate analyses, multivariable modeling identified age and body temperature as the only independent predictors of disease severity, suggesting that oral findings should be interpreted as supplementary clinical indicators rather than determinants of severity.

Nevertheless, oral manifestations remain clinically relevant in dental practice, as they may facilitate early suspicion of varicella infection, particularly before the appearance of the characteristic exanthema. Their recognition supports appropriate patient triage, postponement of elective dental procedures, and implementation of standard infection control measures in suspected cases to reduce the risk of transmission within dental settings.

### Limitations

This study has several limitations that should be acknowledged. First, its retrospective design relies on the accuracy and completeness of existing medical records, which may have contributed to underreporting of oral manifestations, particularly in outpatient settings. Second, hospitalized and outpatient cases were drawn from different healthcare settings, which could introduce some differences between the groups, although both were from the same geographic area and study period. Third, the relatively small sample size, especially in some clinical subgroups, may limit the generalizability of the findings, reduce statistical power, and affect the stability of regression estimates. As this was a retrospective study based on available medical records, a priori sample size calculation was not feasible. Data on comorbidities and complications were limited and inconsistently documented, preventing evaluation of potential confounding effects. Finally, the absence of dynamic follow-up prevented assessment of the temporal evolution of oral lesions in relation to disease severity. These limitations should be considered when interpreting the results.

## 5. Conclusions

Oral manifestations of varicella are a component of the clinical presentation of the disease and may contribute to the overall clinical assessment of infection severity. In this study, oral lesions, particularly oral enanthema, were more frequently observed in hospitalized patients and were associated with a more severe clinical course in univariate analyses. However, multivariable analysis identified age and body temperature as the only independent predictors of hospitalization, indicating that oral findings should be considered supplementary clinical indicators rather than independent predictors of disease severity.

Timely recognition and documentation of oral manifestations is important for early clinical suspicion, comprehensive patient assessment, and interdisciplinary management. In dental practice, awareness of these findings may support appropriate infection control measures, postponement of elective procedures, provision of supportive care, and timely referral in suspected cases. Further prospective studies are needed to better define the independent clinical role of oral manifestations in varicella.

## Figures and Tables

**Table 1:** Distribution of clinical manifestations in the total sample.

Clinical Manifestations		*n*	%
Temperature	afebrile: below 37 °C	50	34.7
	subfebrile: 37 °C to 38 °C	59	41.0
	Febrile: 38 °C to 39 °C	27	18.8
	high fever: above 39 °C	7	4.9
	hyperpyrexia	1	0.7
	Total	144	100.0
Rash	maculo-papulo-vesicular	113	78.5
	vesicles + pustules	29	20.1
	vesicles + pustules + hemorrhage	1	0.7
	crusts	1	0.7
	Total	144	100.0
Oral cavity	no enanthema	102	70.8
	enanthema	41	28.5
	aphthae	1	0.7
	Total	144	100.0
Throat	not hyperemic	16	11.1
	hyperemic	128	88.9
	Total	144	100.0
Tongue	no	97	67.4
	dry, coated	26	18.1
	moist, coated	21	14.6
	Total	144	100.0

**Table 2:** Association between the type of treatment and the demographic and clinical characteristics of the patients.

Demographic and Clinical Characteristics of Patients		Treatment		Total *n* (%)	*p*
		Outpatient Treatment *n*(%)	Hospital Treatment *n*(%)		
Gender	male	58 (71.6)	23 (28.4)	81 (100.0)	0.282
	female	39 (61.9)	24 (38.1)	63 (100.0)	
	total	97 (67.4)	47 (32.6)	144 (100.0)	
Temperature	afebrile: below 37 °C	37 (74.0)	13 (26.0)	50 (100.0)	<0.001
	subfebrile: 37 °C to 38 °C	50 (84.7)	9 (15.3)	59 (100.0)	
	Febrile: 38 °C to 39 °C	10 (37.0)	17 (63.0)	27 (100.0)	
	high fever: above 39 °C	0 (0.0)	7 (100.0)	7 (100.0)	
	hyperpyrexia	0 (0.0)	1 (100.0)	1 (100.0)	
	total	97 (67.4)	47 (32.6)	144 (100.0)	
Rash	maculo-papulo-vesicular	81 (71.7)	32 (28.3)	113 (100.0)	0.070
	vesicles + pustules	16 (55.2)	13 (44.8)	29 (100.0)	
	vesicles + pustules + hemorrhage	0 (0.0)	1 (100.0)	1 (100.0)	
	crusts	0 (0.0)	1 (100.0)	1 (100.0)	
	total	97 (67.4)	47 (32.6)	144 (100.0)	
Oral cavity	no enanthema	76 (74.5)	26 (25.5)	102 (100.0)	0.010
	enanthema	21 (51.2)	20 (48.8)	41 (100.0)	
	aphthae	0 (0.0)	1 (100.0)	1 (100.0)	
	total	97 (67.4)	47 (32.6)	144 (100.0)	
Throat	not hyperemic	9 (56.3)	7 (43.8)	16 (100.0)	0.397
	hyperemic	88 (68.8)	40 (31.3)	128 (100.0)	
	total	97 (67.4)	47 (32.6)	144 (100.0)	
Tongue	no	71 (73.2)	26 (26.8)	97 (100.0)	0.026
	dry, coated	17 (65.4)	9 (34.6)	26 (100.0)	
	moist, coated	9 (42.9)	12 (57.1)	21 (100.0)	
	total	97 (67.4)	47 (32.6)	144 (100.0)	
Age (mean ± SD)		5.67 ± 3.145	16.45 ± 17.938	9.19 ± 11.656	<0.001

**Table 3:** Results of binary logistic regression of the simplified model.

		B	S.E.	Wald	df	Sig.	Exp(B)	95% C.I. for EXP(B)	
								Lower	Upper
Step 1 ^a^	Age	0.116	0.032	13.262	1	<0.001	1.123	1.055	1.195
	Temperature	1.046	0.267	15.298	1	<0.001	2.845	1.685	4.805
	Constant	−3.885	0.681	32.515	1	<0.001	0.021		

^a^. Variable(s) entered on step 1: age, temperature.

## Data Availability

The data presented in this study are available upon request from the corresponding author for privacy reasons.

## Supplementary Materials

The following supporting information can be downloaded at: https://www.mdpi.com/article/10.3390/life16040673/s1, Supplementary File S1. Checklist for recording oral symptoms and general indicators related to the severity of the clinical status in patients with Varicella.
