# Supplementary material for: Oral Manifestations of Varicella and Their Contribution to Clinical Assessment in Hospitalized and Outpatient Patients
Source: Life (Basel). 2026 Apr 15;16(4):673. doi: 10.3390/life16040673 (PMC13117907; doi:10.3390/life16040673)
Supplement: Supplementary file 1 [file life-16-00673-s001.zip › life-4236405-supplementary.pdf]

Supplementary file S1

Checklist for recording oral symptoms and general indicators related to the severity of the clinical status in patients with Varicella

Varicella (Chickenpox) Clinical Severity Assessment Checklist

Case Code: ________________________ **Date:** /______

**1. Demographic Data**	
**Gender:**	
• Male	☐
• Female	☐
**Age:** ________ years	
**2. Overall Clinical Severity (Objective State)**	
• **Mild:** Minimal systemic symptoms, limited rash.	☐
• **Moderately Severe:** Significant discomfort, widespread rash, fever.	☐
• **Severe:** Profuse rash, high fever, significant systemic involvement, or signs of complications.	☐
**3. Body Temperature**	
• **Afebrile:** < 37.0°C	☐
• **Subfebrile:** 37.1°C – 38.0°C	☐
• **Febrile:** 38.1°C – 39.0°C	☐
• **High Febrile:** 39.1°C – 40.0°C	☐
• **Hyperpyrexia:** > 40.0°C	☐
**4. Cutaneous Rash Assessment**	
• **Maculo-Papulo-Vesicular Rash** (Classic Varicella lesions)	☐
• **With Pustules** (Secondary bacterial infection suspected)	☐

• **With Hemorrhagic Components** (Hemorrhagic varicella)	☐
• **Presence of Crusts** (Healing stage)	☐
**5. Oral Mucosa Examination (Enanthem)**	
• **Enanthem Present**	☐
• **No Enanthem Observed**	☐
• **Aphthae / Ulcerations Present**	☐
**6. Oropharyngeal Examination**	
• **Hyperemic** (Inflamed, red)	☐
• **Not Hyperemic** (Normal appearance)	☐
**7. Tongue Assessment**	
• **Dry, Coated**	☐
• **Moist, Coated**	☐
• **No Pathological Changes Registered**	☐
**8. Documented Complications**	
**9. Relevant Comorbidities**	
